# Neuroendocrine Disruption of Reproduction by Bisphenols and Phthalates: From Molecular Mechanisms to Reproductive Outcomes

**DOI:** 10.3390/ijms27167311

**Published:** 2026-08-16

**Authors:** Codruța Claudia Gherman Lencu, Cezara Andreea Gerdanovics, Olga Hilda Orășan, Dana Monica Iancu, Alexandru Gerdanovics, Mirela Georgiana Perne, Mircea Vasile Milaciu, Vasile Negrean, Ioana Raluca Dobrotă, Teodora Gabriela Alexescu

**Affiliations:** 1 Department of Endocrinology-Fundamental Medicine, Iuliu Hatieganu University of Medicine and Pharmacy, Victor Babeș Street No. 8, 400347 Cluj-Napoca, Romania; codruta_lmc@yahoo.com; 2 4th Department of Internal Medicine, 4th Medical Discipline, Iuliu Hatieganu University of Medicine and Pharmacy, Republicii Street No. 18, 400015 Cluj-Napoca, Romania; 3 Department 1, Fundamental Discipline, Anatomy, Iuliu Hatieganu University of Medicine and Pharmacy, Victor Babeș Street No. 8, 400347 Cluj-Napoca, Romania; 4 Neuroscience Department, Iuliu Hatieganu University of Medicine and Pharmacy, 400012 Cluj-Napoca, Romania; 5 Clinical Rehabilitation Hospital, Viilor Street, No. 46-50, 400066 Cluj-Napoca, Romania

**Keywords:** bisphenols, phthalates, endocrine-disrupting chemicals, reproductive toxicity, fertility, steroidogenesis, oxidative stress, epigenetics

## Abstract

Bisphenols and phthalates are ubiquitous endocrine-disrupting chemicals with potential effects on reproductive neuroendocrine regulation, particularly during sensitive developmental windows. This review summarizes current evidence on their exposure profile, mechanisms of reproductive disruption, and female- and male-specific reproductive adversities. A structured narrative review was conducted using PubMed/MEDLINE, Scopus, and Web of Science, including experimental, epidemiological, biomonitoring, systematic review, and meta-analytic studies addressing bisphenols, phthalates, fertility, hormonal regulation, gametogenesis, pregnancy outcomes, and reproductive toxicity mechanisms. Bisphenols and phthalates interfere with estrogenic and androgenic signaling, hypothalamic–pituitary–gonadal axis regulation, steroidogenesis, oxidative stress, inflammation, apoptosis, mitochondrial function, and epigenetic regulation. Reported outcomes include impaired ovarian function, altered oocyte development, hormonal imbalance, reduced semen quality, defective spermatogenesis, sperm DNA damage, infertility, and adverse developmental or pregnancy-related effects. However, human evidence remains heterogeneous and limited by observational designs, exposure misclassification, residual confounding, and insufficient mixture assessment. Current evidence supports biological plausibility for bisphenol- and phthalate-related reproductive toxicity, but causal inference remains limited. Prospective studies with repeated biomonitoring, standardized reproductive endpoints, sex- and age-specific analyses, and improved mixture modeling are needed. A precautionary reduction in avoidable exposure appears reasonable, particularly during sensitive reproductive and developmental periods.

## 1. Introduction

Endocrine-disrupting chemicals (EDCs) are exogenous compounds capable of interfering with hormonal synthesis, secretion, transport, receptor binding, signaling, and metabolism. Among these, bisphenols and phthalates are of particular concern because they are widely used in plastics, food-contact materials, personal care products, medical devices, and other consumer goods, resulting in continuous exposure across the life course [1,2].

EDCs can adversely affect the reproductive system and related cellular processes in both sexes, potentially contributing to congenital abnormalities and impaired fertility [3,4,5,6,7,8]. However, the mechanisms underlying their pathophysiological effects in humans have not yet been fully elucidated [9]. Although an increasing number of studies have investigated the relationship between couples’ exposure to non-persistent EDCs and fecundability, evidence linking biomarker-confirmed exposure to time to pregnancy remains limited and inconclusive. Nevertheless, these uncertainties do not eliminate concerns regarding potential reproductive harm, particularly given the documented endocrine-disrupting effects of these substances on other reproductive outcomes and fetal development. Although persistent organic pollutants and pesticides have historically contributed important evidence regarding reproductive endocrine disruption, the present review focuses specifically on bisphenols and phthalates because of their widespread contemporary exposure and relevance to reproductive health [10,11]. Declining fertility indicators reported in several populations are likely multifactorial, but increasing exposure to environmental toxicants has been proposed as one potentially contributing factor to impaired reproductive function in both men and women [11,12,13].

Human exposure occurs through dietary, inhalational, dermal, occupational, medical, and maternal–fetal routes, with exposure beginning as early as prenatal life. Reducing avoidable exposure to non-persistent EDCs has therefore been recommended, particularly among couples planning pregnancy [14,15,16,17].

EDCs interfere with androgen- and estrogen-mediated pathways by altering hormone synthesis, metabolism, transport, receptor binding, and signaling, thereby contributing to reproductive and developmental abnormalities. Evidence from in vivo, in vitro, in silico, and epidemiological studies supports adverse reproductive effects associated with exposure to several EDC classes, although the strength of these associations varies according to study design and exposure assessment [11].

Recent reviews have addressed important aspects of endocrine-disrupting chemicals and reproductive toxicity, including broad mechanistic frameworks and phthalate-focused reproductive effects. However, the incremental contribution of the present review lies in its integrated bisphenol-plus-phthalate perspective and in its emphasis on how these two widely encountered classes of non-persistent endocrine-disrupting chemicals may converge on reproductive neuroendocrine regulation, gonadal steroidogenesis, oxidative and epigenetic injury, mixture effects, and female- and male-specific reproductive adversities. Unlike reviews focused mainly on single chemical classes or narrower mechanistic domains, this manuscript brings together exposure and toxicokinetic considerations, receptor-level and non-genomic signaling, hypothalamic–pituitary–gonadal axis disruption, critical developmental windows, male and female reproductive outcomes, mixture assessment, and public-health implications within a single molecular and reproductive framework. The central question addressed by this review is how bisphenols and phthalates, as two widely encountered classes of non-persistent endocrine-disrupting chemicals, may converge on reproductive neuroendocrine regulation and molecular pathways of reproductive toxicity, and how strongly these mechanisms are supported by experimental and human evidence. Therefore, the purpose of this review is not only to summarize exposure profiles and reproductive outcomes, but also to compare mechanistic and epidemiological findings, identify areas of convergence and contradiction, and clarify the main uncertainties that currently limit causal interpretation. Particular emphasis is placed on receptor-mediated and non-genomic signaling, hypothalamic–pituitary–gonadal axis regulation, gonadal steroidogenesis, oxidative and epigenetic injury, critical windows of susceptibility, combined exposure and mixture effects, methodological limitations, and public-health implications.

## 2. Materials and Methods

### 2.1. Review Design and Search Strategy

A structured narrative review was conducted to critically evaluate the molecular, cellular, endocrine, and neuroendocrine mechanisms through which bisphenols and phthalates disrupt reproductive function and to integrate experimental and human evidence regarding female and male reproductive outcomes. Particular attention was given to chemical-mixture effects, susceptible developmental windows, methodological limitations, and future research priorities.

A comprehensive literature search was performed in PubMed/MEDLINE, Scopus, and Web of Science Core Collection for articles published in English from database inception to June 2026. The search combined controlled vocabulary, where available, and free-text terms related to bisphenols, phthalates, endocrine disruption, and reproductive health. The principal keywords included “endocrine-disrupting chemicals”, “bisphenol A”, “bisphenol S”, “bisphenol F”, “bisphenol analogues”, “phthalates”, “phthalate metabolites”, “reproductive toxicity”, “fertility”, “infertility”, “fecundability”, “ovarian function”, “spermatogenesis”, “semen quality”, “steroidogenesis”, “pregnancy outcomes”, “developmental exposure”, “neuroendocrine regulation”, “oxidative stress”, “inflammation”, “epigenetic mechanisms”, “low-dose effects”, “non-monotonic dose response”, and “chemical mixtures”. Search syntax was adapted to the requirements of each database. The reference lists of relevant original articles, systematic reviews, and meta-analyses were also manually screened to identify additional eligible publications. Foundational mechanistic studies were retained when they provided essential information on receptor signaling, reproductive neuroendocrine regulation, steroidogenesis, toxicokinetics, or developmental programming, while recent studies and reviews were prioritized for current evidence on reproductive outcomes, biomonitoring, mixture effects, and emerging methodological approaches.

The search process was iterative, as appropriate for a structured narrative review. Multiple combinations of the predefined search terms were used across databases in order to capture mechanistic, toxicological, epidemiological, biomonitoring, and review literature relevant to bisphenols, phthalates, endocrine disruption, reproductive toxicity, and reproductive neuroendocrine regulation. Because the review was not designed as a systematic review and the searches were not performed as a single PRISMA-oriented search string, a formal numerical record flow was not generated. Therefore, numerical counts of records identified, screened, excluded, and included are not reported, in order to avoid presenting retrospectively reconstructed figures that could not be verified with certainty.

### 2.2. Eligibility Criteria and Study Selection

Original in vitro and in vivo studies, observational human studies, biomonitoring investigations, systematic reviews, and meta-analyses were considered eligible when they examined exposure to bisphenols, phthalates, or their metabolites in relation to reproductive development, hormonal regulation, gametogenesis, fertility, pregnancy-related outcomes, or biologically plausible mechanisms of reproductive toxicity. Studies addressing prenatal, childhood, pubertal, preconception, pregnancy, and adult exposure were included, as were investigations of low-dose exposure, replacement bisphenols, chemical mixtures, and sex-specific effects.

Articles were excluded when they did not specifically assess bisphenols or phthalates, focused exclusively on unrelated toxicological outcomes, provided insufficient information on exposure or outcome assessment, or consisted of conference abstracts, editorials, commentaries, letters, or duplicate publications. Non-English publications were also excluded.

Retrieved records were screened by title and abstract by two reviewers, followed by full-text evaluation of potentially relevant articles. Disagreements regarding inclusion were resolved by discussion and, when necessary, by consultation with a third author. Reference lists of relevant original articles, systematic reviews, meta-analyses, and recent narrative reviews were also manually examined in order to identify additional studies providing distinct mechanistic, toxicological, epidemiological, or biomonitoring information.

### 2.3. Data Extraction and Evidence Synthesis

Relevant information was extracted regarding study design, population or experimental model, sample size, chemical investigated, exposure assessment, biological matrix, dose or concentration, exposure timing, reproductive outcomes, mechanistic pathways, principal findings, and major methodological limitations. The evidence was organized according to chemical class, biological mechanism, sex-specific reproductive effects, developmental window of exposure, and study type.

Greater interpretative weight was assigned to studies with prospective designs, repeated exposure measurements, validated analytical methods, appropriate control groups, biologically relevant exposure levels, and adequate adjustment for confounding factors. Experimental evidence was primarily used to assess biological plausibility and mechanistic pathways, whereas human studies were used to evaluate the relevance of these effects under real-world exposure conditions. Observational associations were interpreted cautiously and were not considered definitive evidence of causality.

Because of substantial heterogeneity in exposure metrics, biological matrices, study populations, experimental models, and reproductive outcomes, a quantitative meta-analysis was not performed. Findings were therefore synthesized narratively, with attention to the consistency, direction, and biological plausibility of the reported associations, including positive, null, and conflicting results. Specific consideration was given to exposure misclassification resulting from the short biological half-lives of bisphenols and phthalates, the limitations of single biomarker measurements, non-monotonic dose–response relationships, contamination during sample collection or analysis, and the complexity of combined chemical exposure. In the revised synthesis, evidence was therefore discussed according to whether findings were relatively consistent, partially consistent, or inconsistent across experimental and human studies.

PRISMA guidelines and a formal PRISMA flow diagram were not applied because the present article was designed as a structured narrative review rather than a systematic review. Nevertheless, methodological transparency was strengthened by reporting the databases searched, the main search concepts, eligibility criteria, exclusion criteria, screening process, reviewer involvement, full-text assessment, and use of reference-chaining and hand-searching. This approach was intended to improve reproducibility while preserving the narrative and integrative scope of the review.

## 3. Exposure Profile and Biological Characteristics of Bisphenols and Phthalates

### 3.1. Sources and Routes of Human Exposure

Bisphenols and phthalates are widely used in consumer, industrial, medical, and food-contact materials. Their widespread use results in continuous low-level exposure through ingestion, inhalation, dermal absorption, occupational contact, medical procedures, and maternal–fetal transfer. Dietary exposure is particularly relevant for BPA and several phthalates because of their use in food-contact materials, packaging, and processing environments, whereas dermal and inhalational exposure are especially relevant for phthalates used in personal care products, indoor materials, and household dust [18,19,20,21,22,23,24,25].

BPA is primarily used as a monomer in polycarbonate plastics, epoxy resins lining food and beverage containers, and thermal paper products. Degradation of BPA-containing polymers may facilitate chemical migration, particularly under conditions of elevated temperature, prolonged use, or mechanical stress. DEHP, a high-molecular-weight phthalate, is widely used as a plasticizer to increase the flexibility and elasticity of polyvinyl chloride products, including food packaging and medical devices. Because phthalates are not covalently bound to the polymer matrix, they can leach, volatilize, or migrate into food, air, dust, and biological fluids during product use. Their widespread application results in continuous exposure through multiple routes, and biomonitoring studies have detected BPA and phthalate metabolites in urine, blood, cord blood, and other biological matrices in exposed populations [20]. This section is intended to summarize exposure sources and routes only; reproductive associations and mechanistic evidence are discussed separately in the sections on toxicokinetics, molecular mechanisms, and female- and male-specific reproductive adversities.

### 3.2. Bisphenol Families and BPA Substitutes

Bisphenols comprise a broad family of structurally related chemicals used primarily in the manufacture of polycarbonate plastics and epoxy resins, including food and beverage packaging, dental sealants, thermal paper, medical devices and toys. BPA remains the most extensively used and studied member of this group and is present in a wide range of consumer products [19]. In 2017, the European Chemicals Agency included BPA in the Candidate List of Substances of Very High Concern because of its endocrine-disrupting properties and potential adverse effects on reproductive, metabolic, and other biological functions [20].

Regulatory restrictions and increasing public concern regarding BPA have encouraged its replacement with structurally similar analogs, particularly BPS and BPF, as well as BPAF, BPB, BPAP, and several less frequently used compounds. These substitutes generally share physicochemical and structural characteristics with BPA and may therefore interact with similar endocrine pathways.

However, their biological effects remain considerably less well characterized, and available toxicological evidence has focused predominantly on their endocrine-disrupting activity. Although BPS and BPF were introduced as alternatives to BPA, accumulating evidence indicates that these analogs may retain endocrine-disrupting properties and should not automatically be regarded as biologically inert or safer substitutes [19].

### 3.3. Phthalate Families and Metabolism

Phthalates are widely used as plasticizers in polyvinyl chloride products and in numerous consumer, pharmaceutical, and medical applications. High-molecular-weight compounds such as DEHP, DiNP, and DiDP are primarily used to impart flexibility to PVC materials, whereas lower-molecular-weight phthalates, including DMP, DEP, DBP, and DiBP, are more commonly found in personal care products, coatings, adhesives, pharmaceuticals, and other consumer goods. Because phthalates are not covalently bound to the polymer matrix, they can migrate into food, water, air, dust, and materials that come into direct contact with the skin [20].

Human exposure occurs mainly through ingestion, inhalation, and dermal absorption. Dietary sources include contaminated dairy products, fish, seafood, oils, and food-contact materials, while inhalation exposure may result from indoor and outdoor air, household dust, and emissions near manufacturing facilities. Dermal exposure is particularly relevant for phthalates used in personal care products. Infants and fetuses may also be exposed through placental transfer, breast milk, and mouthing of phthalate-containing toys or other consumer products [21,22,23,24,25]. Owing to their widespread use, environmental dispersion, and continuous release from consumer materials, exposure to phthalates is considered nearly ubiquitous.

### 3.4. Toxicokinetic and Exposure-Assessment Considerations

Phthalates are rapidly metabolized after entering the human body and generally have short biological half-lives, often within several hours [26]. Their metabolism initially involves hydrolysis of the parent diester into the corresponding monoester metabolite, followed by oxidation and phase II conjugation, predominantly glucuronidation mediated by uridine 5′-diphospho-glucuronosyltransferases. The resulting hydrophilic metabolites are eliminated mainly through urine, although fecal and, to a lesser extent, sweat excretion may also occur [20].

The metabolic pathway varies according to molecular structure. Low-molecular-weight phthalates are primarily hydrolyzed to monoesters and subsequently excreted in urine, whereas high-molecular-weight compounds undergo additional oxidative transformations before conjugation and elimination [27,28]. For example, DEHP is initially converted to mono(2-ethylhexyl) phthalate (MEHP) and subsequently to several oxidative metabolites, including mono(2-ethyl-5-hydroxyhexyl) phthalate, mono(2-ethyl-5-oxohexyl) phthalate, mono(2-ethyl-5-carboxypentyl) phthalate (MECPP), and mono(2-carboxymethylhexyl) phthalate (MCMHP). Because oxidative metabolites are generally more abundant and may better reflect cumulative DEHP exposure than MEHP alone, urinary MECPP and related metabolites are widely used as biomarkers in epidemiological studies [28].

Age and physiological status may influence phthalate metabolism. Children have been reported to excrete a greater proportion of oxidative DEHP metabolites relative to MEHP than adults, indicating age-related differences in biotransformation [29]. Placental transfer and the detection of metabolites in maternal and fetal biological samples further support exposure during sensitive developmental periods [20].

These toxicokinetic features have important implications for exposure assessment, because single urinary measurements often reflect recent rather than habitual exposure. Repeated sampling and the use of multiple metabolites are therefore preferable in studies evaluating reproductive outcomes [20,30,31,32,33].

Reported biomonitoring concentrations of bisphenols and phthalate metabolites vary widely according to population, age, pregnancy status, geographical region, sampling matrix, timing of collection, analytical method, and exposure source [20,22,26,28,29,30,31,32,33,34,35,36]. In human studies, BPA, BPS, BPF, and phthalate metabolites are most commonly assessed in urine, although serum, cord blood, maternal or fetal biological samples, semen, placenta, and breast milk have also been used in specific reproductive or developmental settings [20,30,31,32,33,35,36]. For phthalates, urinary monoester and oxidative metabolites, such as MEHP, MECPP, MCMHP, MBP, and MiBP, are generally preferred over parent compounds because they better reflect internal exposure and reduce the risk of external contamination [20,28,29,32,34]. However, because bisphenols and phthalates are non-persistent compounds with short biological half-lives, single biomarker concentrations usually reflect recent exposure rather than long-term internal dose [26,28,29,32]. These limitations are discussed further in the Critical Appraisal section, particularly in relation to exposure misclassification, concentration–outcome interpretation, and the absence of robust clinically applicable exposure thresholds.

Because individuals are exposed simultaneously to multiple phthalates with partly overlapping antiandrogenic and reproductive effects, compound-by-compound assessment can underestimate overall risk. Cumulative approaches based on hazard quotients and hazard indices may therefore provide a more appropriate framework for evaluating combined exposure, particularly among pregnant women, infants, and other susceptible populations [35].

Regarding bisphenols, BPA, BPS, and BPF are among the most frequently detected compounds in human biomonitoring studies, indicating widespread exposure in the general population. Although BPS and BPF are generally detected at lower concentrations than BPA, their increasing use as replacement chemicals has raised concerns regarding continuous population exposure. Compared with BPA, however, considerably less is known about their absorption, distribution, metabolism, elimination, and long-term biological effects [36].

The detection of BPS and BPF in cord blood provides evidence that these compounds may cross the placenta and reach the fetal compartment. Experimental studies further suggest that gestational or perinatal exposure to BPA substitutes may alter placental endocrine function, mammary gland development, neurobehavioral outcomes, and reproductive or metabolic programming, although the relevance of these findings to typical human exposure remains uncertain. Because the placenta regulates pregnancy maintenance, endocrine signaling, and fetal nutrient and oxygen exchange, disruption of placental homeostasis may have consequences for both pregnancy progression and offspring development [36].

Toxicokinetic evidence for BPA substitutes remains limited, particularly during pregnancy. Available human studies have largely involved non-pregnant participants, while physiological changes during gestation may alter chemical distribution, metabolism, and elimination. Pregnancy-specific physiologically based toxicokinetic models must account for the maternal, placental, amniotic, and fetal compartments, but their development is currently constrained by limited in vivo data for BPS, BPF, and other emerging analogs [36]. These uncertainties reinforce the need for improved biomonitoring and pregnancy-specific toxicokinetic studies before BPA substitutes can be considered safer alternatives. The principal sources of exposure, toxicokinetic characteristics, biomarkers, and reproductive effects of the major bisphenols and phthalates discussed in this review are summarized in Table 1.

## 4. Neuroendocrine Regulation of Reproduction and Critical Windows of Susceptibility

### 4.1. The Hypothalamic–Pituitary–Gonadal Axis

The hypothalamic–pituitary–gonadal axis coordinates reproductive development and function through the integrated regulation of gonadotropin secretion, gonadal steroidogenesis, gametogenesis, and sex-specific reproductive processes. Estrogens and androgens are central components of this regulatory network and contribute to gonadal development, secondary sexual characteristics, menstrual-cycle regulation, ovarian function, and spermatogenesis. Although estrogen concentrations differ between sexes, estradiol is biologically relevant in both females and males and contributes to reproductive-organ development and the regulation of spermatogenesis [37,38,39]. Androgens, particularly testosterone and dihydrotestosterone, are essential for male reproductive development and function.

Because the hypothalamic–pituitary–gonadal axis depends on tightly regulated estrogenic and androgenic feedback, bisphenols and phthalates can disrupt reproductive function by altering receptor signaling, gonadotropin regulation, steroidogenesis, and gametogenesis. These mechanisms are discussed in detail in the following section [40,41,42,43,44,45,46]. Metabolic alterations, including insulin resistance and changes in aromatase activity, may further modulate neuroendocrine feedback, although their precise contribution to impaired human reproductive function remains incompletely established [47].

Overall, disruption of estrogenic and androgenic signaling by BPA and phthalates may alter feedback regulation across the hypothalamic, pituitary, and gonadal levels. Such interference can affect gonadotropin secretion, steroid-hormone balance, gonadal development, and gametogenesis, ultimately contributing to reproductive dysfunction in both sexes.

### 4.2. Critical Developmental Windows

The biological consequences of exposure to bisphenols and phthalates depend not only on dose but also on the timing of exposure. Prenatal, fetal, neonatal, and early postnatal life represent particularly sensitive periods because endocrine signaling is essential for sexual differentiation, gonadal development, reproductive-tract maturation, and the long-term programming of reproductive function [48,49,50,51,52]. Disruption during these stages can therefore produce effects that persist into adulthood.

Prenatal exposure to EDCs has been associated with altered sexual development in both male and female offspring and may contribute to reproductive disorders later in life [50,51,52]. BPA is of particular concern during fetal and neonatal development because it may interfere with estrogenic signaling and induce lasting reproductive changes [53,54]. Maternal exposure has been linked experimentally to abnormal reproductive-organ development, impaired ovarian function, altered oocyte quality, and disrupted spermatogenesis in the offspring [42,43]. Transgenerational effects have also been reported, with male descendants appearing especially susceptible in some experimental models [42].

Phthalate exposure during development may similarly affect both male and female fetuses. Experimental evidence indicates that developmental exposure can alter ovarian development, increase uterine size, reduce fertility, and impair androgen-dependent reproductive differentiation [55]. Because phthalates may inhibit androgen-receptor signaling and reduce gonadal steroidogenesis, exposure during periods of active reproductive-tissue differentiation may be particularly harmful [46,50,51,52,53,54,55,56].

Maternal–fetal transfer represents an important route of developmental exposure. Sex hormone-binding globulin limits placental transfer of endogenous maternal hormones, whereas certain EDCs, including BPA, display lower binding affinity and may therefore remain biologically available [57]. Exposure may also continue after birth through breastfeeding, which has been associated with the transfer of endocrine-active compounds during another vulnerable period of reproductive maturation [58].

Although developmental stages are generally considered the most susceptible, adult exposure may also contribute to subfertility, infertility, and hormonal imbalance [58]. Nevertheless, the available evidence supports the concept that exposure during critical developmental windows may have more profound and persistent effects than exposure occurring after reproductive maturation.

## 5. Mechanisms of Reproductive Neuroendocrine Disruption

### 5.1. Interaction with Nuclear and Membrane Hormone Receptors

A central mechanism of reproductive endocrine disruption involves the ability of bisphenols and phthalates to interact with nuclear and membrane-associated hormone receptors. These compounds can act as receptor agonists or antagonists, thereby modifying the transcriptional and non-transcriptional actions of endogenous estrogens and androgens. Estrogen signaling is mediated primarily through ERα and ERβ, which, following ligand binding, translocate to the nucleus and interact with estrogen response elements to regulate target-gene transcription. Estrogenic signals may also be transmitted through membrane-bound estrogen receptors and G protein-coupled receptors, activating downstream pathways involving EGFR, IGFR, GPCRs, NF-κB, AP-1, MAPK, PKA, Akt, Erk, and PAK [59,60,61].

At the molecular level, the endocrine-disrupting activity of bisphenols and phthalates depends not only on their ability to bind hormone receptors, but also on the receptor subtype involved, ligand-binding domain conformation, recruitment of co-regulators, and activation of downstream transcriptional or non-genomic signaling pathways. BPA has lower affinity for classical nuclear estrogen receptors than endogenous 17β-estradiol, but it can still activate ERα- and ERβ-dependent responses at low concentrations and may also interact with estrogen-related receptor γ. Structural and computational studies suggest that BPA binding involves specific interactions within receptor ligand-binding domains, supporting the concept that even relatively weak receptor binding may produce biologically relevant signaling effects when exposure occurs during sensitive developmental windows [40,41,62,63,64,65].

BPA can further activate membrane-associated pathways through G protein-coupled receptors and GPR30. At very low concentrations, BPA has been reported to activate PKA- and PKG-dependent signaling through GPCRs, influencing germ-cell proliferation and differentiation [62]. Through GPR30-related MAPK signaling, BPA has also been reported to promote apoptosis in reproductive cells, including spermatocytes [63]. These non-genomic mechanisms may help explain how biologically relevant effects occur even when affinity for classical nuclear estrogen receptors is limited.

Beyond estrogen receptors, BPA may interact with androgen and progesterone receptors. In silico analyses have identified several amino-acid residues involved in BPA binding to the androgen receptor and progesterone receptor, suggesting that these interactions may interfere with androgen-dependent reproductive development and progesterone-mediated processes required for female reproductive-tract function and pregnancy maintenance [64]. Nuclear magnetic resonance studies have further suggested that BPA may interact with the N-terminal domain of the androgen receptor [65].

For phthalates, molecular docking and receptor-activation studies indicate that several parent compounds and metabolites interact with ERα, ERβ, and AR, with predominantly antagonistic effects on androgen signaling. Reported AR interactions involve hydrophobic contacts within the ligand-binding domain, including residues such as Phe764, Leu704, Asn705, and Thr877. These structural interactions reduce androgen-dependent transcriptional activity and thereby affect genes involved in Leydig-cell function, Sertoli-cell support, testicular development, and spermatogenesis [44,66].

Overall, the ability of bisphenols and phthalates to interact with both nuclear and membrane hormone receptors enables them to disrupt reproductive signaling at multiple levels. These interactions may modify receptor activation, intracellular kinase cascades, transcription-factor activity, and the expression of genes involved in reproductive development and function.

### 5.2. Disruption of Hypothalamic and Pituitary Signaling

Bisphenols and phthalates may interfere with reproductive neuroendocrine regulation by altering the signaling pathways that connect the hypothalamus and pituitary gland with the gonads. Estrogenic and androgenic signals contribute to the feedback mechanisms that regulate gonadotropin secretion, gonadal steroidogenesis, and gametogenesis. Consequently, disruption of these pathways may impair the coordinated hormonal control required for normal reproductive function [59,60,61].

At the hypothalamic level, reproductive function is primarily coordinated by the pulsatile secretion of gonadotropin-releasing hormone (GnRH), which controls pituitary luteinizing hormone (LH) and follicle-stimulating hormone (FSH) release. GnRH pulsatility is regulated by upstream neuronal networks, particularly kisspeptin-expressing neurons and the arcuate kisspeptin/neurokinin B/dynorphin, or KNDy, neuronal system. These neuronal populations integrate sex-steroid feedback, metabolic signals, developmental cues, and environmental influences, making them biologically plausible targets for endocrine-disrupting chemicals.

BPA may modify neuroendocrine signaling through both nuclear and membrane-associated estrogen pathways. By mimicking 17β-estradiol and activating ER-dependent signaling, BPA alters transcriptional responses involved in reproductive regulation [40]. It may also act through GPCR- and GPR30-mediated pathways, activating PKA, PKG, and MAPK signaling at very low concentrations [62,63]. Because these intracellular cascades participate in hormone-responsive cellular processes, their inappropriate activation may disturb the transmission of estrogenic signals within the reproductive axis.

These effects may be particularly relevant for kisspeptin-mediated regulation of GnRH secretion, because kisspeptin neurons are highly sensitive to sex-steroid feedback and play a central role in controlling the timing, frequency, and amplitude of GnRH pulses. Therefore, disruption of estrogenic or androgenic signaling by bisphenols and phthalates may indirectly alter kisspeptin tone, GnRH pulsatility, and downstream gonadotropin secretion. During prenatal, neonatal, and pubertal windows, such interference may also affect the organization of sexually dimorphic neuroendocrine circuits involved in pubertal timing, reproductive behavior, and long-term hypothalamic–pituitary–gonadal axis function.

Alterations in metabolic and hormonal feedback may provide an additional mechanism. Exposure to BPA and phthalates has been proposed to reduce gonadotropin secretion through pathways associated with insulin resistance [47]. Furthermore, increased aromatase activity elevates estradiol levels and disrupts downstream hypothalamic–pituitary signaling, thereby affecting the hormonal environment required for spermatogenesis [47]. These effects suggest that EDC-related neuroendocrine disruption can occur not only through direct receptor binding but also through secondary changes in metabolic and steroid hormone homeostasis.

Phthalates also impair signaling along the reproductive axis through their predominantly antagonistic effects on the androgen receptor [44,66]. Reduced androgen-receptor activity can weaken androgen-dependent feedback and compromise the transcriptional regulation of genes involved in gonadal function. In experimental models, phthalate exposure has been associated with reduced testosterone and estradiol production, together with decreased expression of steroidogenic factor-1 and specificity protein-1, indicating disruption of the molecular signals that support gonadal steroidogenesis [56].

At the pituitary level, bisphenols and phthalates may influence gonadotroph function indirectly by altering GnRH input, sex-steroid feedback, and peripheral steroidogenesis. Potential downstream consequences include altered LH and FSH secretion, impaired gonadal steroid production, and disrupted gametogenesis. However, direct evidence regarding gonadotroph-specific effects remains less developed than evidence for receptor-mediated, steroidogenic, oxidative, and epigenetic mechanisms in gonadal tissues.

Although the available evidence supports interference with hormonal feedback and downstream signaling, direct evidence regarding specific effects on hypothalamic GnRH neurons, kisspeptin/KNDy networks, or pituitary gonadotrophs remains limited. Therefore, the proposed effects on hypothalamic and pituitary regulation should be interpreted mainly as consequences of altered estrogenic, androgenic, metabolic, and steroidogenic signaling rather than as fully characterized direct toxic effects on these structures. Future studies should specifically assess GnRH pulsatility, kisspeptin/KNDy signaling, gonadotroph responsiveness, LH and FSH secretion patterns, and sex-specific neurodevelopmental programming after developmental and adult exposure to bisphenols, phthalates, and their mixtures.

### 5.3. Altered Gonadal Steroidogenesis

Altered gonadal steroidogenesis represents a key downstream consequence of receptor-mediated endocrine disruption. Interference with steroid hormone synthesis may alter testosterone, estradiol, and other hormones required for sexual differentiation, gametogenesis, and fertility [44,46,56].

At the molecular level, phthalate-induced impairment of steroidogenesis has been linked to altered expression of transcriptional and enzymatic regulators required for testosterone and estradiol biosynthesis. Experimental studies indicate that dibutyl and monobutyl phthalates may reduce the expression of steroidogenic factor-1 (SF-1) and specificity protein-1 (Sp-1), two transcriptional regulators involved in Leydig-cell steroidogenic activity [56]. Disruption of this regulatory network may affect the expression or activity of key steroidogenic components, including steroidogenic acute regulatory protein, cholesterol side-chain cleavage enzyme, 3β-hydroxysteroid dehydrogenase, 17β-hydroxysteroid dehydrogenase, and aromatase, thereby reducing androgen production and disturbing estrogen–androgen balance. These molecular changes provide a mechanistic link between receptor-level antiandrogenic effects, impaired Leydig-cell function, altered gonadotropin feedback, and defective spermatogenesis [44,46,56].

BPA can influence gonadal hormone production indirectly through estrogenic and antiandrogenic receptor activity. By mimicking 17β-estradiol and interacting with estrogen, androgen, and progesterone receptors, BPA can disturb the hormonal balance required for normal ovarian and testicular function [40,64]. Experimental evidence indicates that adult BPA exposure impairs ovarian function and oocyte development, whereas in males it has been associated with adverse effects on spermatogenesis [42,43]. Compared with phthalates, however, the available evidence is less direct for BPA-specific effects on steroidogenic enzyme expression or activity. Developmental exposure may further amplify these effects. Transgenerational phthalate exposure has been associated with abnormal ovarian development, increased uterine size, and reduced fertility in female offspring [55]. These findings suggest that altered steroidogenesis during critical developmental periods contributes not only to immediate hormonal disturbances but also to persistent reproductive abnormalities later in life.

### 5.4. Oxidative Stress, Inflammation, and Mitochondrial Dysfunction

Oxidative stress represents a major mechanism through which endocrine-disrupting chemicals may impair reproductive function. Reactive oxygen species (ROS), including oxygen-derived free radicals and non-radical oxidants, are normally generated during cellular metabolism but may damage lipids, proteins, and nucleic acids when their production exceeds antioxidant defenses. Spermatozoa are particularly susceptible because of their limited cytoplasmic antioxidant capacity and reduced ability to repair DNA damage. Excessive oxidative stress therefore compromises sperm motility, fertilizing capacity, and genomic integrity, with potential consequences for embryo development [67,68,69]. BPA has been reported to increase intracellular reactive oxygen species and induce DNA strand breaks, thereby contributing to defective spermiogenesis and impaired sperm function [70,71]. Epidemiological findings have also suggested an association between urinary BPA concentrations and markers of DNA damage, although direct evidence linking these alterations to clinically relevant reproductive outcomes remains limited [71]. Because estrogenic signaling can modulate cellular redox balance, disruption of estrogen-dependent pathways may further increase reproductive-cell vulnerability to oxidative injury [59,60,61,67,68,69,70,71]. The molecular consequences of receptor-mediated disruption also involve activation of intracellular kinase and stress-response pathways. BPA-related activation of GPR30-associated MAPK signaling has been linked to apoptosis in spermatocytes, while GPCR-mediated activation of PKA and PKG may alter germ-cell proliferation and differentiation [62,63]. In parallel, increased reactive oxygen species may induce DNA strand breaks, impair sperm chromatin integrity, and interact with methylation-dependent gene regulation. These changes suggest convergence between non-genomic estrogen signaling, oxidative DNA damage, and apoptosis-related pathways in reproductive cells [67,68,69,70,71].

Phthalate exposure has also been associated with oxidative injury in reproductive tissues, particularly in experimental models of male reproductive toxicity. Increased ROS production contributes to germ-cell damage, disruption of the blood–testis barrier, mitochondrial dysfunction, apoptosis, and impaired spermatogenesis. These effects are consistent with the broader antiandrogenic and steroidogenic disruption described above, although phthalate-specific oxidative mechanisms remain less extensively characterized than receptor-mediated and steroidogenic effects [44,46,56].

Oxidative stress can also interact with broader epigenetic and transcriptional mechanisms. In germ cells, these changes may affect meiosis, chromosome stability, spermatogenesis, and oocyte competence [62,63,67,68,69,70,71]. Inflammatory signaling and mitochondrial dysfunction may further amplify reproductive toxicity. Activation of stress-responsive pathways, including MAPK, Akt, NF-κB, and AP-1, may promote cytokine production, pro-apoptotic signaling, and disruption of cellular homeostasis in gonadal tissues. Mitochondria are particularly relevant because steroidogenesis, oocyte maturation, sperm motility, and germ-cell survival depend on adequate mitochondrial function and redox balance. Excessive ROS production impairs mitochondrial membrane potential, disturbs ATP generation, promotes mitochondrial-dependent apoptosis, and aggravates DNA and chromatin damage. BPA-mediated activation of GPR30-related MAPK signaling has been associated with apoptosis in spermatocytes, indicating that disrupted non-genomic signaling may converge with oxidative and mitochondrial injury to promote reproductive-cell loss [62,63,67,68,69,70,71]. Overall, the available evidence supports oxidative stress and related DNA damage as biologically plausible contributors to bisphenol- and phthalate-associated reproductive toxicity, particularly in experimental reproductive-cell and male germ-cell models. However, the extent of compound-specific evidence differs across BPA, phthalates, sexes, tissues, and endpoints. The precise roles of inflammation and mitochondrial dysfunction also remain less clearly characterized in the current literature and require further mechanistic investigation.

**Figure 1:**
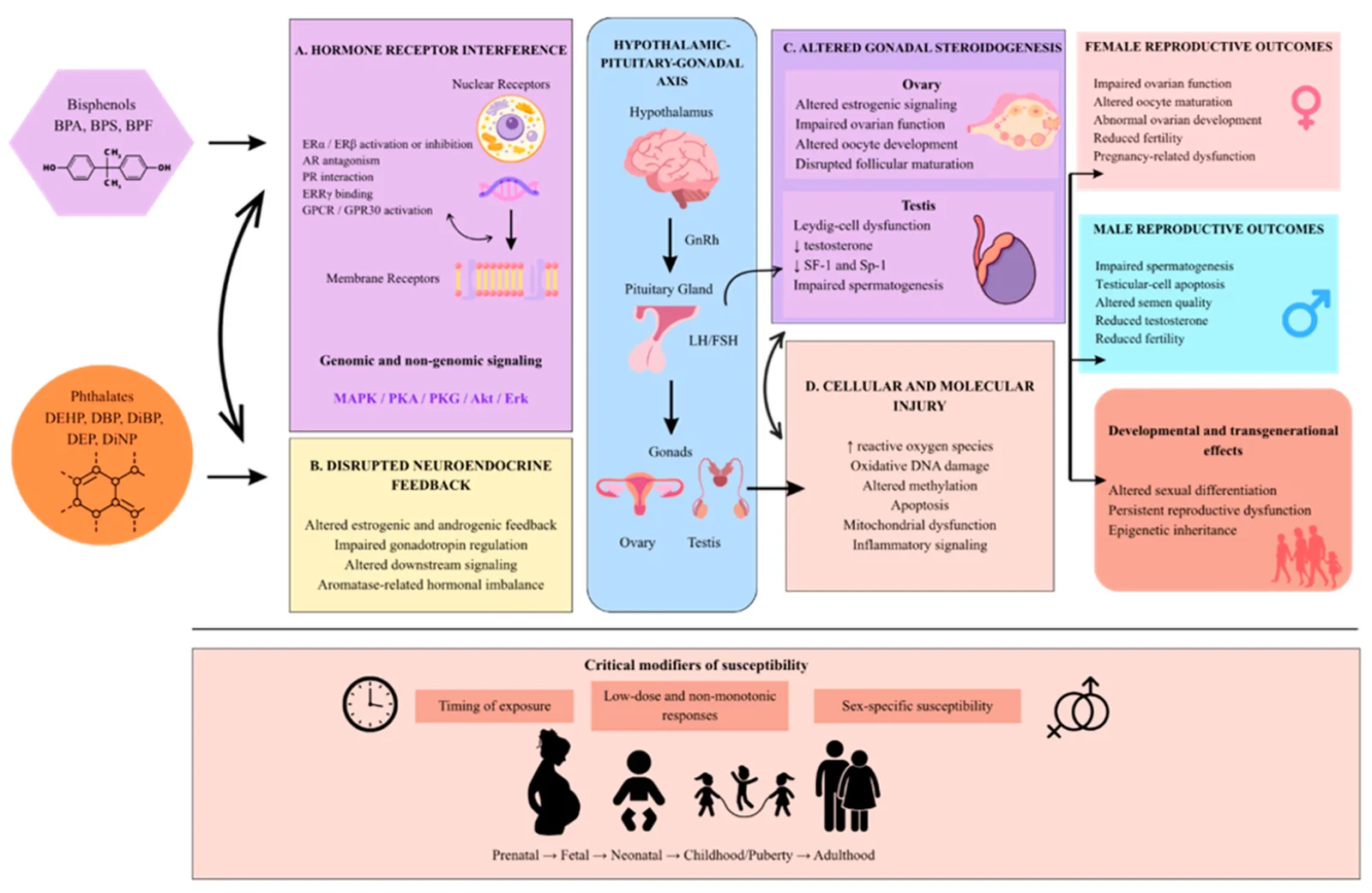
Graphical representation of the proposed molecular and neuroendocrine mechanisms of bisphenol- and phthalate-induced reproductive disruption. Bisphenols may activate or interfere with ERα, ERβ, ERRγ, GPR30, GPCR-associated PKA/PKG signaling, MAPK activation, oxidative stress, DNA damage, apoptosis, and epigenetic remodeling. Phthalates and their metabolites may antagonize AR signaling, interact with ERα/ERβ, impair Leydig-cell steroidogenic regulators such as SF-1 and Sp-1, reduce steroid hormone synthesis, disrupt blood–testis barrier integrity, and alter DNA methylation and microRNA-related pathways. These molecular events converge on altered GnRH/gonadotropin feedback, impaired steroidogenesis, defective gametogenesis, and female- and male-specific reproductive adversities. Figure 2 summarizes the main female- and male-specific reproductive adversities associated with bisphenol and phthalate exposure, including receptor-mediated disruption, altered steroidogenesis, oxidative stress, apoptosis, epigenetic remodeling, and downstream effects on ovarian, follicular, oocyte-related, Leydig-cell, spermatogenic, semen-quality, and sperm DNA integrity endpoints.

The principal molecular and neuroendocrine mechanisms through which bisphenols and phthalates may disrupt reproductive function are summarized graphically in Figure 1. These compounds can interfere with nuclear and membrane hormone receptors, alter hypothalamic–pituitary–gonadal signaling, impair gonadal steroidogenesis, and promote oxidative and molecular injury. The resulting effects may compromise gametogenesis, gonadal function, fertility, and reproductive development, while their magnitude may depend on the dose, timing of exposure, developmental stage, and biological sex.

**Figure 2:**
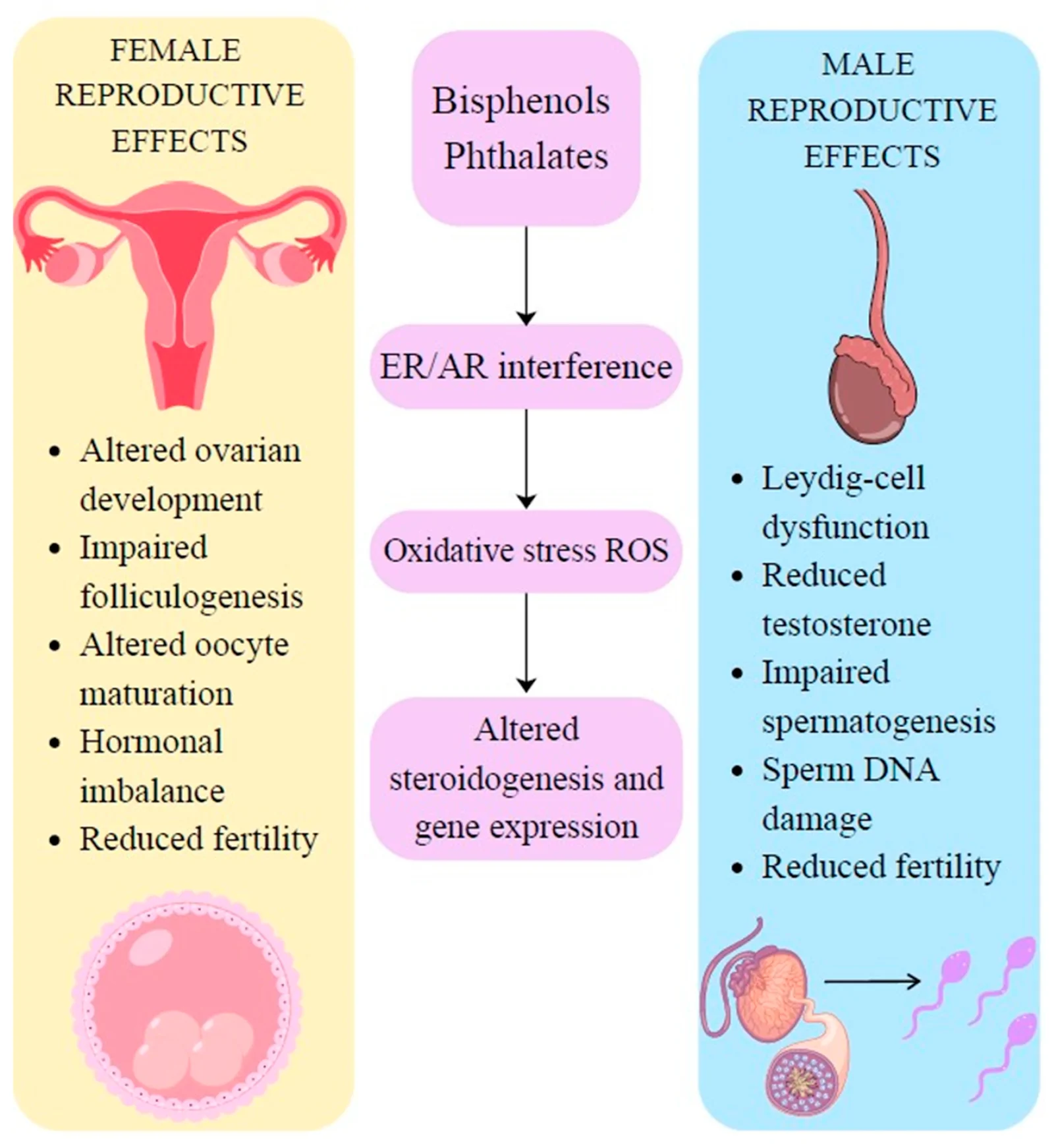
Female- and male-specific reproductive adversities associated with bisphenol and phthalate exposure. The figure summarizes the alignment between steroid-receptor interference, altered steroidogenesis, oxidative stress, apoptosis, epigenetic remodeling, and reproductive outcomes affecting ovarian function, follicular survival, oocyte competence, Leydig-cell function, spermatogenesis, semen quality, and sperm DNA integrity.

### 5.5. Epigenetic and Intergenerational Effects

Endocrine-disrupting chemicals are increasingly recognized as potential inducers of epigenetic alterations, providing a biologically plausible mechanism through which reproductive effects may persist across generations. When exposure occurs during pregnancy, the pregnant individual (F0), the developing fetus (F1), and the fetal germ cells that may later give rise to the F2 generation are simultaneously exposed. Effects observed in these directly exposed generations are therefore considered multigenerational, whereas effects occurring in the F3 generation represent true transgenerational inheritance because this is the first generation not directly exposed to the original chemical [72].

Growing experimental evidence suggests that several EDCs induce multigenerational or transgenerational changes in reproductive endpoints, including pubertal timing, fertility, fecundity, reproductive behavior and parental behavior [73,74,75]. These effects may be mediated through persistent alterations in DNA methylation, histone modifications, and the expression or activity of long non-coding RNAs and microRNAs, although the underlying mechanisms remain incompletely characterized [76].

More specific epigenetic alterations have been reported in relation to both bisphenol and phthalate exposure. These include changes in DNA methylation patterns, altered expression of DNA methyltransferases, global methylation changes, and dysregulation of non-coding RNAs, including microRNAs and long non-coding RNAs [76,77,78,79,80,81,82,83,84,85,86,87,88,89]. BPA exposure has been associated with altered placental microRNA expression, reduced global DNA methylation in zebrafish reproductive models, and methylation-related changes in spermatocytes [80,90,91]. Phthalate exposure has similarly been associated with differential DNA methylation in cord blood, imprinted genes, and conserved non-genic elements, as well as heritable methylation changes affecting male reproductive development [87,88,89]. These findings support epigenetic remodeling as a molecular mechanism through which transient developmental exposure may produce persistent reproductive effects.

Evidence in humans remains limited, heterogeneous, and sometimes inconsistent, largely because transgenerational effects require long-term follow-up across several generations and are difficult to distinguish from shared environmental, behavioral, and socioeconomic influences [77]. Experimental evidence suggests that developmental exposure to bisphenols and phthalates induces persistent epigenetic alterations affecting reproductive development, steroidogenesis, gametogenesis, and fertility later in life [80,81,82,83,84,85,86,87,88,89]. Accordingly, epigenetic reprogramming and its potential contribution to intergenerational and transgenerational reproductive dysfunction remain important priorities in current EDC research.

Because reproductive toxicity manifests differently in females and males, the term “sex-specific reproductive outcomes” is used here to refer to female- and male-specific reproductive adversities and their mechanistic links to steroid-receptor signaling, steroidogenesis, oxidative injury, apoptosis, and epigenetic regulation. These relationships are summarized in Table 2.

## 6. Female Reproductive Outcomes

### 6.1. Experimental Evidence

Exposure to phthalates has been associated with multiple adverse effects on female reproductive function, involving ovarian structure, follicular survival, steroidogenesis, and reproductive aging. Experimental studies in female rodents have reported reduced ovarian weight and estradiol concentrations, increased follicular atresia, and depletion of the ovarian reserve. These outcomes are consistent with the oxidative, inflammatory, apoptotic, and steroidogenic mechanisms discussed above [11].

In vitro evidence further indicates that phthalate metabolites, particularly mono(2-ethylhexyl) phthalate (MEHP), may compromise granulosa-cell viability, steroidogenic activity, and follicular growth through multiple cellular stress pathways. Developmental exposure may produce persistent reproductive abnormalities, as experimental phthalate exposure has been associated with abnormal ovarian development, increased uterine size, and reduced fertility in female offspring [55]. Oxidative stress also contributes to female reproductive toxicity by impairing granulosa-cell function, follicular survival, oocyte competence, and ovarian steroidogenesis. In ovarian tissue, excessive ROS production promotes lipid, protein, and DNA damage, activates apoptotic pathways, and disturbs the redox-sensitive signaling required for follicular maturation. These mechanisms provide a plausible link between bisphenol- or phthalate-related molecular injury and outcomes such as follicular atresia, altered estradiol production, impaired oocyte quality, and reduced ovarian reserve [55,79,80,81,82,83,84,85].

Bisphenol exposure may likewise impair female reproductive function through estrogen-receptor-mediated, epigenetic, and cellular mechanisms. Adult BPA exposure has been associated experimentally with abnormal ovarian function and impaired oocyte development [42]. BPA can also disrupt oocyte competence and ovarian function through altered aromatase regulation, microRNA expression, and epigenetic remodeling [80,81,82,83,84,85]. Nevertheless, human findings remain heterogeneous, and causal interpretation is limited by differences in exposure assessment, age, reproductive status, and study design.

### 6.2. Human Epidemiological and Clinical Evidence

Human epidemiological studies provide partial support for these experimental observations by linking urinary phthalate metabolites with altered estradiol and gonadotropin concentrations, reduced ovarian reserve, infertility, and changes in reproductive timing. Associations with follicle-stimulating hormone and anti-Müllerian hormone may be nonlinear and appear to vary according to age. For example, phthalate exposure has been associated with a lower antral follicle count among women younger than 35 years, whereas different or opposite patterns have been reported in older women. Exposure during development also influences pubertal timing, with prolonged exposure being associated with earlier pubertal onset in females [79].

Nevertheless, human findings remain heterogeneous, and causal interpretation is limited by differences in exposure assessment, age, reproductive status, biological matrix, timing of sampling, and study design. Therefore, experimental findings support biological plausibility, whereas human studies currently provide mainly associative evidence rather than definitive causal proof.

## 7. Male Reproductive Outcomes

### 7.1. Experimental Evidence

Male reproductive toxicity associated with phthalate exposure is characterized by structural testicular injury, impaired steroidogenesis, disruption of spermatogenesis, and deterioration of sperm quality. Animal studies have shown that DEHP, DBP, BBP, and their mixtures may produce dose-dependent reductions in testicular weight, degeneration of the seminiferous epithelium, and impairment of the blood–testis barrier. The latter has been linked to reduced expression of tight-junction and adhesion proteins, including ZO-1, CX-43, and N-cadherin. These structural alterations are accompanied by decreased expression of spermatogenic proteins, reduced sperm concentration and motility, and increased morphological abnormalities [11,79].

These findings are consistent with the antiandrogenic and steroidogenic mechanisms described above, particularly impaired androgen-receptor signaling, Leydig-cell dysfunction, and reduced testosterone synthesis [44,46,56,66].

Oxidative stress further contributes to phthalate-associated testicular toxicity by damaging germ cells, disrupting the blood–testis barrier, and activating cell-death pathways. Highly lipophilic phthalates such as DnOP accumulate in spermatozoa and inhibit phospholipase A2, an enzyme involved in the acrosome reaction required for fertilization. Epigenetic mechanisms may also contribute: DEHP has been shown to alter DNA methyltransferase expression and produce heritable methylation changes affecting male reproductive development [86,87,88,89].

BPA can impair male reproductive function through estrogenic, antiandrogenic, oxidative, and epigenetic pathways. Experimental evidence links BPA exposure with impaired spermatogenesis, sperm DNA damage, altered meiotic processes, and disrupted spermatogenic gene regulation [11,90,91,92,93,94,95,96,97].

### 7.2. Human Epidemiological and Clinical Evidence

Human epidemiological studies have associated phthalate and bisphenol exposure with reduced semen quality, hormonal imbalance, and infertility, although results remain inconsistent. Urinary phthalate metabolites have been inversely associated with testosterone concentrations and with sperm concentration, motility, and morphology. Exposure during early or late puberty may also influence adult semen quality, emphasizing that age and developmental timing may modify male reproductive susceptibility. Prospective studies using repeated exposure measurements and clinically relevant fertility outcomes remain necessary to clarify these relationships [11].

Overall, experimental studies provide stronger mechanistic evidence for testicular, steroidogenic, spermatogenic, oxidative, and epigenetic disruption, whereas human studies mainly support associations with semen quality, reproductive hormones, and infertility-related endpoints. These human findings should therefore be interpreted cautiously because of exposure misclassification, within-person variability in semen parameters, residual confounding, and the predominantly observational nature of the available data.

## 8. Combined Exposure and Chemical-Mixture Effects

Humans are simultaneously exposed to multiple phthalates, bisphenols, and other endocrine-disrupting chemicals, making single-compound assessment an incomplete representation of real-world exposure. Chemicals within the same class can act through overlapping pathways, including estrogen- and androgen-receptor interference, suppression of steroidogenesis, oxidative stress, inflammatory activation, apoptosis, and epigenetic dysregulation [44,56,62,63,67,68,69,70,71,98,99]. However, the strength of evidence for mixture effects differs across endpoints and study types. Experimental data, particularly for male reproductive toxicity, provide support for combined effects on steroidogenesis, testicular development, spermatogenesis, oxidative injury, and epigenetic regulation [98,99]. In contrast, human and female-specific mixture evidence remains more limited and heterogeneous, because most available studies rely on biomonitoring-based co-exposure patterns, single time-point measurements, and variable reproductive endpoints. Importantly, mixture studies should distinguish between chemicals sharing a common adverse-outcome pathway and broader exposome-level analyses, because the interpretation and regulatory implications differ substantially.

For the female reproductive system, mixture-related effects are biologically plausible because bisphenols and phthalates converge on estrogen-receptor signaling, ovarian steroidogenesis, granulosa-cell function, follicular survival, oocyte competence, oxidative stress, and epigenetic regulation. Epidemiological and biomonitoring studies also suggest associations between combined exposure profiles and reproductive hormones, ovarian reserve markers, pubertal timing, infertility-related endpoints, and pregnancy-related outcomes. Nevertheless, mixture-specific evidence for female reproductive outcomes remains less consistent than experimental evidence for male reproductive toxicity, and causal interpretation remains limited by exposure misclassification, correlated exposures, residual confounding, and heterogeneous outcome definitions. Experimental studies indicate that mixtures of DEHP, DBP, BBP, and other phthalates produce dose-dependent testicular injury, impairment of the blood–testis barrier, reduced testosterone synthesis, and deterioration of sperm quality. Because several phthalates share antiandrogenic properties, their combined effects may accumulate even when individual exposure levels are relatively low. Similarly, concurrent exposure to bisphenols and phthalates may disrupt both estrogenic and androgenic signaling, potentially amplifying hormonal imbalance across the hypothalamic–pituitary–gonadal axis [44,56,62,63,67,68,69,70,71,98,99].

Mixture exposure may also influence epigenetic regulation. Chronic exposure to EDC mixtures has been associated with altered microRNA expression and post-transcriptional dysregulation of genes involved in testicular cell death and spermatogenic failure [98]. Experimental exposure to DEHP combined with other endocrine-active compounds has likewise produced differential methylation of genes involved in reproductive and developmental pathways [99]. These findings suggest that mixture toxicity may extend beyond receptor-level interactions and include persistent molecular reprogramming.

Epidemiological assessment of chemical mixtures remains challenging because phthalate and bisphenol biomarkers are frequently correlated, exposure levels vary over time, and conventional regression models may not adequately capture nonlinear or interactive effects. Cumulative risk approaches, including hazard-index methods and modern mixture-analysis techniques, may therefore be more appropriate than evaluating each chemical independently. However, the interpretation of combined exposure remains limited by heterogeneous mixture compositions, short-lived biomarkers, single-sample exposure assessment, and insufficient longitudinal evidence. Future studies should incorporate repeated biomonitoring, sex- and age-stratified analyses, and statistical methods specifically designed to evaluate complex chemical mixtures.

## 9. Critical Appraisal of the Available Evidence

The available evidence supports the biological plausibility of reproductive toxicity associated with bisphenol and phthalate exposure, but its strength varies considerably across experimental, mechanistic, and epidemiological studies. A major strength of the literature is the convergence of findings from in vitro, in vivo, in silico, animal, cellular, and human biomonitoring studies. However, the supporting evidence differs across mechanisms, compounds, biological models, exposure windows, and reproductive endpoints. To improve readability and clarify the weight of evidence supporting the main conclusions of this review, the principal evidence domains are summarized in Table 3. The table reports representative supporting literature, biological model or study type, exposure context, major findings, and interpretation of evidence consistency. Exposure route or exposure period is reported where available; for receptor-binding, in silico, review-based, or indirect mechanistic sources, exposure context is described according to the source type rather than inferred. 

Based on this structured evidence mapping, the strongest support is provided by experimental and mechanistic evidence for receptor-mediated signaling, phthalate-related steroidogenic disruption, oxidative injury, apoptosis, and epigenetic remodeling. Female- and male-specific reproductive adversities are biologically plausible and partially supported by human biomonitoring and epidemiological studies, but the consistency of findings varies according to compound, sex, exposure route, exposure timing, biological matrix, endpoint, and study design. Direct hypothalamic and pituitary effects, replacement bisphenols, human mixture effects, concentration–outcome thresholds, and true transgenerational inheritance in humans remain comparatively less established.

Partially consistent evidence is observed for female- and male-specific reproductive adversities. Experimental studies provide stronger support for altered ovarian development, impaired oocyte competence, Leydig-cell dysfunction, impaired spermatogenesis, sperm DNA damage, and altered semen-related endpoints. Human biomonitoring and epidemiological studies support possible associations with reproductive hormones, ovarian reserve markers, semen quality, pubertal timing, infertility-related endpoints, and pregnancy outcomes, but these associations are more heterogeneous and are influenced by exposure timing, biological matrix, age, reproductive status, and study design. Inconsistent or insufficient evidence remains particularly important for direct hypothalamic and pituitary effects, concentration–outcome correlations, mixture effects in humans, replacement bisphenols, and transgenerational effects in human populations. For these areas, the available literature includes positive, null, nonlinear, and sometimes conflicting findings. These discrepancies are likely explained by the short biological half-lives of bisphenols and phthalates, reliance on single urinary measurements, differences in timing of exposure assessment, heterogeneous reproductive endpoints, residual confounding, and limited longitudinal data. Therefore, the current evidence supports biological plausibility and concern but does not yet allow robust causal inference or clinically applicable exposure thresholds. The evidence is particularly persuasive when similar effects are observed across multiple biological levels. For example, phthalate-induced antiandrogenic activity has been demonstrated through androgen-receptor inhibition, impaired Leydig-cell function, reduced expression of steroidogenic regulators, and decreased testosterone production [44,46,56,66]. Likewise, BPA has been shown to interfere with classical and membrane-associated estrogen pathways, increase reactive oxygen species, alter DNA integrity, and disrupt epigenetic regulation in reproductive tissues [67,68,69,70,71]. Such convergence strengthens the biological plausibility of the reported reproductive outcomes.

Nevertheless, several limitations restrict causal interpretation. Much of the human evidence is derived from cross-sectional or case–control studies, which are vulnerable to reverse causality, residual confounding, and uncertainty regarding the temporal relationship between exposure and reproductive dysfunction. The cause–effect relationship between BPA exposure and male fertility, for example, remains difficult to establish because semen parameters show substantial within- and between-individual variability and are often assessed concurrently with exposure biomarkers [100].

Exposure assessment represents another major source of uncertainty. Bisphenols and phthalates generally have short biological half-lives, and concentrations measured in a single urine sample can reflect only recent exposure rather than usual or long-term exposure. Within-person variability, differences in urine dilution, timing of sample collection, analytical methodology, and potential contamination from plastic laboratory materials lead to exposure misclassification. Studies using repeated biological sampling and standardized analytical procedures are therefore more informative than those relying on isolated measurements [44,46,56,62,63,67,68,69,70,71,80,81,82,83,84,85,86,87,88,89,98,99]. The interpretation of concentration–outcome relationships is also limited by the heterogeneity of reported biomarker concentrations, biological matrices, timing of sampling, statistical adjustment, reproductive endpoints, and effect estimates. Although several epidemiological studies have reported associations between urinary bisphenol or phthalate metabolite concentrations and reproductive hormones, ovarian reserve markers, semen quality parameters, pubertal timing, infertility-related endpoints, or pregnancy outcomes, the reported effect sizes, correlation coefficients, and dose–response patterns are not directly comparable across studies [17,26,79,100]. Therefore, the available evidence does not currently support robust concentration thresholds or clinically applicable predictive cut-offs. Future studies should report standardized biomarker concentrations, use repeated sampling, evaluate non-monotonic dose–response relationships, and provide comparable effect estimates for reproductive outcomes.

Experimental studies also have important limitations. Animal and in vitro models permit detailed investigation of receptor signaling, tissue morphology, gene expression, and cellular injury, but administered doses may exceed typical environmental exposure levels. Differences in species, metabolism, developmental timing, route of administration, and reproductive physiology further complicate translation to humans. Some experimental outcomes, including structural testicular damage, follicular atresia, and marked changes in steroidogenic enzymes, may therefore not directly correspond to the magnitude or pattern of effects observed under real-world exposure conditions.

Outcome heterogeneity is another concern. Female studies assess diverse endpoints, including estradiol concentrations, antral follicle count, anti-Müllerian hormone, follicular atresia, oocyte quality, pubertal timing, infertility, and pregnancy outcomes. Male studies similarly vary in their evaluation of testosterone, semen concentration, motility, morphology, sperm DNA integrity, testicular histology, and fertility. Differences in age, reproductive status, exposure window, laboratory methods, and statistical adjustment reduce comparability across studies and may partly explain inconsistent findings.

Several apparent contradictions can be explained by differences in exposure window, biological matrix, timing of biomarker collection, sex, age, reproductive status, and endpoint definition. Developmental exposure may produce effects that differ from adult exposure, while single urinary measurements do not accurately represent the biologically relevant exposure period. Similarly, associations with ovarian reserve markers, gonadotropins, testosterone, semen parameters, or pubertal timing can differ according to age and baseline reproductive status. These factors explain why some studies report adverse associations whereas others report weak, null, nonlinear, or even opposite findings.

The interpretation of hormonal outcomes is particularly complex because endocrine-disrupting chemicals produce nonlinear and sex-specific effects. Associations with follicle-stimulating hormone, anti-Müllerian hormone, testosterone, or estradiol may differ according to age, developmental stage, dose, and biological sex. Findings observed in younger women may not be reproduced in older populations, while exposure during prenatal development, puberty, or adulthood may result in distinct reproductive phenotypes. Consequently, pooled interpretations that do not account for age and exposure timing may obscure biologically meaningful differences.

Mixture exposure further limits the relevance of conventional single-chemical studies. Humans are simultaneously exposed to multiple bisphenols, phthalates, and other endocrine-active substances, many of which affect overlapping pathways. Correlated exposures, shared metabolites, and potential additive, synergistic, or antagonistic effects are difficult to separate using standard regression models. Although experimental mixture studies support cumulative antiandrogenic, oxidative, inflammatory, and epigenetic effects, human evidence remains comparatively limited and methodologically heterogeneous [44,46,56,62,63,67,68,69,70,71,80,81,82,83,84,85,86,87,88,89,98,99].

Finally, epigenetic and transgenerational findings should be interpreted cautiously. Alterations in DNA methylation, histone modifications, and microRNA expression provide plausible mechanisms for persistent reproductive effects, particularly in experimental and developmental models. The evidence supporting epigenetic remodeling and potential intergenerational or transgenerational effects is summarized separately in Table 3. Overall, the current evidence supports concern regarding the reproductive effects of bisphenols and phthalates, particularly during sensitive developmental periods and under repeated or combined exposure. However, stronger causal inference will require prospective human studies with repeated biomonitoring, standardized reproductive endpoints, detailed assessment of developmental timing, improved control of confounding, and analytical approaches capable of evaluating complex chemical mixtures. Greater harmonization between experimental and epidemiological research is also needed to clarify which molecular and cellular effects are most relevant to human fertility and reproductive health.

## 10. Public Health, Regulation, and Exposure-Reduction Strategies

Because bisphenols and phthalates are widely present in food-contact materials, plastics, cosmetics, household products, medical devices, and occupational settings, complete avoidance is unrealistic. Nevertheless, precautionary exposure-reduction measures are justified, particularly for pregnant women, infants, children, individuals undergoing fertility treatment, and couples planning pregnancy [17].

Regulatory restrictions on BPA and selected phthalates have reduced certain sources of exposure, but substitution with structurally related compounds may introduce alternatives whose reproductive safety remains insufficiently characterized [101,102]. Therefore, class-based regulation, improved premarket testing, and transparent labeling may be more effective than restricting individual compounds sequentially.

Practical measures include avoiding the heating or prolonged storage of food in plastic containers, reducing the use of heavily packaged foods, selecting phthalate-free personal care products when possible, improving indoor ventilation and dust control, and applying appropriate occupational protection. In medical settings, safer alternatives to phthalate-containing devices can be considered for vulnerable patients when clinically feasible.

Public-health strategies should also account for critical developmental windows, low-dose and non-monotonic effects, sex-specific susceptibility, and combined exposure to multiple endocrine-active chemicals [11]. Overall, coordinated action involving regulators, manufacturers, healthcare professionals, employers, and consumers is needed to reduce preventable exposure while further research determines whether these interventions improve reproductive outcomes.

## 11. Limitations

Several limitations should be considered when interpreting the evidence synthesized in this review. Although a structured search strategy was applied, this manuscript was designed as a narrative review rather than a systematic review; therefore, PRISMA reporting, protocol registration, formal risk-of-bias assessment, and quantitative synthesis were not performed. In addition, the included literature was highly heterogeneous with respect to exposure metrics, biological matrices, developmental windows, reproductive endpoints, and study designs.

Interpretation of the evidence is further limited by the short biological half-lives of bisphenols and phthalates, frequent reliance on single urinary measurements, residual confounding in observational studies, limited human evidence for replacement compounds and mixture effects, and uncertain translation from experimental models to real-world human exposure. These limitations should be considered when interpreting the conclusions of this review. Future research may benefit from integrating multi-omics and computational approaches to improve the mechanistic interpretation of bisphenol- and phthalate-related reproductive toxicity. Transcriptomics, epigenomics, proteomics, metabolomics, and exposomics may help identify molecular signatures linking exposure biomarkers with oxidative stress, mitochondrial dysfunction, inflammatory activation, steroidogenic disruption, gametogenic defects, and reproductive outcomes. In parallel, adverse-outcome-pathway frameworks, machine-learning models, and neural-network-based approaches may support the prioritization of chemicals for reproductive toxicity testing and help predict pathway-level effects when experimental data are limited. Recent computational studies have shown that machine-learning and deep-neural-network models can be applied to predict chemical reproductive toxicity and female reproductive toxicity pathways using molecular descriptors, bioactivity data, and adverse-outcome-pathway information [103,104,105]. However, these approaches should be considered complementary to experimental and epidemiological research, because their predictive value depends on data quality, biological interpretability, external validation, and integration with clinically relevant reproductive endpoints. In addition, the heterogeneity of reported biomarker concentrations, biological matrices, sampling strategies, and effect estimates prevented a reliable comparison of concentration–outcome correlations across studies.

## 12. Conclusions

Bisphenols and phthalates are widespread endocrine-disrupting chemicals that interfere with reproductive function through receptor-mediated, steroidogenic, oxidative, inflammatory, mitochondrial, and epigenetic pathways. These mechanisms provide biological plausibility for reported associations with impaired ovarian function, altered oocyte development, reduced semen quality, defective spermatogenesis, hormonal imbalance, infertility, and adverse developmental outcomes.

However, the strength of evidence varies across outcomes, chemicals, developmental windows, and study designs. Human data remain limited by exposure misclassification, observational designs, residual confounding, and insufficient evaluation of mixtures and replacement compounds. Future research should prioritize prospective studies with repeated biomonitoring, standardized reproductive endpoints, age- and sex-specific analyses, and advanced mixture modeling. Until stronger causal evidence is available, a precautionary approach to reducing avoidable exposure appears appropriate, particularly during sensitive reproductive and developmental windows.

## Figures and Tables

**Table 1:** Summary of the main bisphenols and phthalates discussed in relation to human exposure and reproductive health, including exposure sources, routes, biomarkers, and principal molecular or reproductive considerations based on the cited exposure, toxicokinetic, biomonitoring, and reproductive literature [18,19,20,21,22,23,24,25,26,28,29,30,31,32,33,34,35,36].

Compound/Class	Main Uses and Exposure Sources	Principal Exposure Routes	Metabolism and Relevant Biomarkers	Main Molecular Effects and Reproductive Outcomes
**BPA** **[18,19,20,21,36]**	Polycarbonate plastics, epoxy resins, food and beverage packaging, thermal paper, dental materials, medical devices, and toys	Dietary ingestion, dermal contact, inhalation, and maternal–fetal transfer	Commonly assessed through urinary BPA; toxicokinetics may be modified during pregnancy	Estrogenic and antiandrogenic signaling, altered ovarian function, impaired oocyte development, spermatogenic disruption, placental effects, and developmental reproductive concerns
**BPS** **[19,36]**	BPA substitute used in thermal paper, plastics, food-contact materials, and consumer products	Dietary, dermal, inhalation, and transplacental exposure	Detected in urine and fetal cord blood; pregnancy-specific toxicokinetic data remain limited	Potential estrogenic activity and altered placental endocrine function; experimental links with fertility disruption, neurodevelopmental changes, and reproductive or metabolic programming
**BPF** **[19,36]**	BPA substitute used in epoxy resins, coatings, and food-contact materials	Dietary, dermal, inhalation, and transplacental exposure	Detected in urine and fetal cord blood, generally at lower concentrations than BPA	Possible endocrine and reproductive effects, including placental and developmental concerns, although human reproductive evidence remains limited
**Other BPA analogues** **[19,36]**	Industrial polymers, resins, and BPA-replacement applications	Mainly dietary, dermal, inhalation, and occupational exposure	Toxicokinetic and biomonitoring data are scarce	Potential receptor-mediated endocrine activity based on structural similarity to BPA; reproductive outcome data remain insufficient
**Low-molecular-weight phthalates, including DMP, DEP, DBP, and DiBP** **[20,21,22,23,24,25,26,28,29,34]**	Personal care products, cosmetics, hair products, pharmaceuticals, adhesives, coatings, and plastics	Dermal absorption, inhalation, and dietary exposure	Primarily hydrolyzed to monoester metabolites, conjugated, and excreted in urine	Altered steroidogenesis, antiandrogenic effects, hormonal imbalance, ovarian and testicular developmental concerns, and possible fertility-related effects
**High-molecular-weight phthalates, including DEHP, DiNP, and DiDP** **[20,21,22,23,24,25,26,28,29,34]**	Flexible PVC, medical tubing, food packaging, flooring, toys, and construction materials	Dietary ingestion, inhalation, dermal contact, occupational exposure, and parenteral exposure from medical devices	Undergo hydrolysis followed by oxidation and glucuronidation; urinary oxidative metabolites are commonly used in biomonitoring	Antiandrogenic and developmental reproductive effects, impaired steroidogenesis, testicular dysfunction, altered semen quality, and pregnancy-related concerns
**DEHP** **[20,26,28,29,34]**	Flexible PVC, food packaging, and medical devices	Dietary, inhalation, dermal, and medical exposure	Metabolized to MEHP and oxidative metabolites, including MECPP and MCMHP; eliminated mainly in urine	Reduced testosterone synthesis, Leydig-cell dysfunction, impaired spermatogenesis, sperm-quality alterations, fetal reproductive development concerns, and adverse pregnancy-related outcomes
**DBP/DiBP** **[20,21,22,23,24,25,26,28,29,34]**	Cosmetics, adhesives, plastics, coatings, and consumer products	Dermal, inhalation, and dietary exposure	Biomarkers include MBP and MiBP in urine	Antiandrogenic and steroidogenic disruption, altered Leydig-cell function, impaired reproductive development, and potential effects on fertility-related endpoints

**Abbreviations:** BPA, bisphenol A; BPS, bisphenol S; BPF, bisphenol F; DMP, dimethyl phthalate; DEP, diethyl phthalate; DBP, dibutyl phthalate; DiBP, diisobutyl phthalate; DEHP, di(2-ethylhexyl) phthalate; DiNP, diisononyl phthalate; DiDP, diisodecyl phthalate; MEHP, mono(2-ethylhexyl) phthalate; MECPP, mono(2-ethyl-5-carboxypentyl) phthalate; MCMHP, mono(2-carboxymethylhexyl) phthalate; MBP, mono-n-butyl phthalate; MiBP, mono-isobutyl phthalate; PVC, polyvinyl chloride.

**Table 2:** Female- and male-specific reproductive adversities associated with bisphenol and phthalate exposure and their mechanistic relevance to steroid-receptor signaling.

Sex/System	Main Reproductive Adversities	Main Compounds/Classes	Receptor-Related and Molecular Relevance
Female reproductive system	Altered ovarian function, impaired oocyte development, altered follicular survival, follicular atresia, reduced ovarian reserve markers, altered estradiol and gonadotropin concentrations, infertility-related endpoints, and changes in pubertal timing	BPA, BPS/BPF, DEHP metabolites, DBP/DiBP, low-molecular-weight phthalates	Mainly linked to altered ERα/ERβ signaling, estrogenic or antiestrogenic activity, disrupted aromatase regulation, altered steroidogenesis, oxidative stress, apoptosis, and epigenetic or microRNA-mediated remodeling [42,55,79,80,81,82,83,84,85]
Pregnancy and developmental reproductive programming	Altered reproductive-tract development, placental endocrine disruption, developmental programming of reproductive function, and potential adverse pregnancy-related outcomes	BPA, BPS, BPF, DEHP, phthalate mixtures	Related to disrupted estrogenic signaling, altered placental function, maternal–fetal transfer, epigenetic programming, and interference with critical developmental windows [36,48,49,50,51,52,53,54,55,72,73,74,75,76,77,78]
Male reproductive system	Leydig-cell dysfunction, reduced testosterone production, impaired steroidogenesis, testicular structural injury, impaired spermatogenesis, reduced sperm concentration and motility, abnormal morphology, and sperm DNA damage	DEHP, DBP, BBP, DnOP, phthalate mixtures, BPA	Mainly linked to AR antagonism, altered ERα/ERβ signaling, reduced SF-1 and Sp-1 expression, impaired steroidogenic enzymes, oxidative stress, mitochondrial dysfunction, apoptosis, blood–testis barrier disruption, and epigenetic alterations [44,46,56,66,79,86,87,88,89]
Neuroendocrine regulation in both sexes	Altered HPG-axis feedback, potential disruption of GnRH/kisspeptin/KNDy signaling, altered LH/FSH secretion patterns, and altered pubertal or reproductive maturation	Bisphenols and phthalates	Related to altered estrogenic and androgenic feedback, receptor-mediated signaling, metabolic-hormonal feedback, gonadotropin regulation, and developmental programming of sexually dimorphic reproductive circuits [40,44,47,56,62,63,66]

**Table 3:** Structured evidence mapping of bisphenol- and phthalate-associated reproductive toxicity, including representative supporting literature, biological model or study type, exposure context, major findings, and interpretation of evidence consistency.

Evidence Domain	Main Supporting Literature	Study Type and Exposure Context	Main Findings	Evidence Interpretation
Receptor-mediated estrogenic and androgenic signaling	[41,44,64,65,66]	In vitro receptor-activation assays, structural/NMR analyses, and in silico docking studies assessing receptor-level interaction with BPA, phthalates, and phthalate metabolites; not designed as exposure-period studies	BPA interacts with estrogen-related, androgen, and progesterone receptors in receptor-level studies. Phthalates and metabolites interact with ERα, ERβ, and AR, with predominantly antagonistic effects on androgen signaling	Relatively consistent mechanistic evidence for receptor interaction, but receptor binding alone does not establish organism-level reproductive toxicity
Non-genomic estrogen signaling and apoptosis	[62,63]	Experimental cellular studies using low-dose BPA exposure in reproductive or germ-cell-related models, including GPCR/PKA/PKG and GPR30/MAPK signaling pathways	BPA activates GPCR-associated PKA/PKG signaling and GPR30/MAPK-related pathways, affecting germ-cell proliferation, differentiation, and apoptosis	Relatively consistent mechanistic evidence, but direct human relevance remains incompletely established
Phthalate-related steroidogenic disruption	[46,49,56]	In vitro and in vivo experimental Leydig-cell and developmental models; exposure contexts include dibutyl/monobutyl phthalate treatment, developmental or in utero exposure, and Leydig-cell steroidogenic assessment	Phthalates reduce testosterone and estradiol production in experimental models and alter steroidogenic regulators such as SF-1 and Sp-1, affecting Leydig-cell function and spermatogenesis	Relatively consistent experimental evidence, especially for male steroidogenesis and antiandrogenic phthalate effects
Oxidative stress, DNA damage, and apoptosis	[62,63,67,68,69,70,71,79]	Mechanistic cellular, reproductive-cell, germ-cell, and human biomonitoring/epidemiological evidence; exposure contexts include low-dose BPA cellular exposure and urinary BPA or phthalate metabolite assessment in human studies	ROS-related injury can damage lipids, proteins, DNA, sperm chromatin, and cellular integrity. BPA has been linked with oxidative DNA damage, while phthalate-related oxidative injury is implicated in germ-cell damage, blood–testis barrier disruption, apoptosis, and impaired spermatogenesis	Biologically plausible and experimentally supported, especially for germ-cell injury; compound-specific human and female-specific evidence remains limited
Epigenetic remodeling and developmental programming	[25,54,80,81,82,83,84,85,86,87,88,89,90,91,97,98,99]	Animal, zebrafish, placental, reproductive-cell, germ-cell, and review evidence; exposure contexts include prenatal, developmental, adult, germ-cell, and transgenerational exposure windows depending on study	Reported mechanisms include altered DNA methylation, DNMT expression, microRNA dysregulation, altered aromatase expression, placental microRNA changes, and possible heritable reproductive effects	Relatively consistent mechanistic evidence in experimental models; true transgenerational inheritance in humans remains insufficiently established
Female reproductive adversities	[42,53,55,58,79,80,81,82,83,84,85]	Animal, in vitro granulosa-cell/oocyte-related, experimental developmental, human epidemiological, and clinical/review evidence; exposure contexts include prenatal, developmental, adult, and urinary biomarker-based exposure	Reported outcomes include abnormal ovarian development, altered oocyte quality, granulosa-cell dysfunction, altered aromatase expression, follicular atresia, ovarian reserve changes, pubertal timing, infertility-related endpoints, and pregnancy-related outcomes	Partially consistent evidence; experimental data support biological plausibility, but human findings vary by age, exposure window, biological matrix, endpoint, and study design
Male reproductive adversities	[43,46,52,56,79,86,87,88,89,90,91,92,93,94,95,96,97,98,99]	Animal, in vitro/in vivo Leydig-cell and germ-cell models, reproductive-cell studies, systematic review evidence, and human epidemiological evidence; exposure contexts include prenatal, pubertal, adult, germ-cell, and urinary biomarker-based exposure	Reported outcomes include Leydig-cell dysfunction, impaired steroidogenesis, reduced testosterone production, altered spermatogenesis, semen-quality changes, sperm DNA damage, methylation changes, and infertility-related endpoints	Partially consistent to relatively consistent evidence, strongest for experimental and mechanistic male reproductive toxicity; human evidence remains affected by exposure misclassification and confounding
Mixture effects	[35,55,98,99]	Experimental phthalate-mixture and EDC-mixture studies, developmental/in utero exposure models, and cumulative-risk assessment approaches; human mixture evidence mainly derives from biomonitoring-based co-exposure patterns	Mixtures can produce cumulative antiandrogenic, steroidogenic, oxidative, inflammatory, and epigenetic effects, particularly in male reproductive models. Reported effects include impaired testicular development, reduced testosterone synthesis, blood–testis barrier injury, altered sperm quality, microRNA dysregulation, and DNA methylation changes	Stronger evidence for experimental male reproductive endpoints; human and female-specific mixture evidence remains limited and heterogeneous
Human exposure–outcome interpretation	[17,20,26,79,99]	Human biomonitoring, epidemiological, systematic review, and meta-analytic evidence; exposure is usually assessed using urinary BPA, BPS, BPF, or phthalate metabolites reflecting recent exposure, with some prenatal, pubertal, preconception, or adult windows	Associations have been reported with reproductive hormones, ovarian reserve markers, semen quality, pubertal timing, infertility-related endpoints, and pregnancy outcomes, but findings vary across populations, matrices, sampling strategies, and endpoints	Partially consistent and heterogeneous; robust concentration thresholds or clinically applicable predictive cut-offs are not established
Direct hypothalamic and pituitary effects	[40,44,47,56,62,63,66,72]	Neuroendocrine review evidence and indirect mechanistic evidence from receptor, metabolic-hormonal, and steroidogenic studies; mainly developmental or mechanistic inference with limited direct human data	Disruption of estrogenic and androgenic feedback can plausibly affect GnRH, kisspeptin/KNDy signaling, LH/FSH regulation, and downstream gonadal function, but direct evidence for hypothalamic or pituitary targets remains limited	Biologically plausible but insufficiently established as a direct toxic effect; evidence remains mainly indirect

## Data Availability

No new data were created or analyzed in this study. Data sharing is not applicable to this article.

## Abbreviations

The following abbreviations are used in this manuscript:

Akt	Protein kinase B
AMH	Anti-Müllerian hormone
AP-1	Activator protein 1
AR	Androgen receptor
BBP	Benzyl butyl phthalate
BPA	Bisphenol A
BPAF	Bisphenol AF
BPAP	Bisphenol AP
BPB	Bisphenol B
BPF	Bisphenol F
BPS	Bisphenol S
CX-43	Connexin 43
DBP	Dibutyl phthalate
DEHP	Di(2-ethylhexyl) phthalate
DEP	Diethyl phthalate
DiBP	Diisobutyl phthalate
DiDP	Diisodecyl phthalate
DiNP	Diisononyl phthalate
DMP	Dimethyl phthalate
DNA	Deoxyribonucleic acid
DnOP	Di-n-octyl phthalate
EDCs	Endocrine-disrupting chemicals
EGFR	Epidermal growth factor receptor
ERα	Estrogen receptor alpha
ERβ	Estrogen receptor beta
Erk	Extracellular signal-regulated kinase
FSH	Follicle-stimulating hormone
GPCRs	G protein-coupled receptors
GPR30	G protein-coupled estrogen receptor 1
HPG axis	Hypothalamic–pituitary–gonadal axis
IGFR	Insulin-like growth factor receptor
LH	Luteinizing hormone
MAPK	Mitogen-activated protein kinase
MBP	Mono-n-butyl phthalate
MCMHP	Mono(2-carboxymethylhexyl) phthalate
MECPP	Mono(2-ethyl-5-carboxypentyl) phthalate
MEHP	Mono(2-ethylhexyl) phthalate
MiBP	Mono-isobutyl phthalate
miRNAs	MicroRNAs
mRNA	Messenger RNA
NF-κB	Nuclear factor kappa B
PAK	p21-activated kinase
PKA	Protein kinase A
PKG	Protein kinase G
PRISMA	Preferred Reporting Items for Systematic Reviews and Meta-Analyses
PVC	Polyvinyl chloride
ROS	Reactive oxygen species
SF-1	Steroidogenic factor 1
Sp-1	Specificity protein 1
ZO-1	Zonula occludens-1
